# Tuning Metamaterials by using Amorphous Magnetic Microwires

**DOI:** 10.1038/s41598-017-09665-5

**Published:** 2017-08-24

**Authors:** V. Lopez-Dominguez, M. A. Garcia, P. Marin, A. Hernando

**Affiliations:** 1 Instituto de Magnetismo Aplicado UCM-adif, A6 km.22’5 – Apdo. Correos 155, Las Rozas, Madrid, 28230 Spain; 2 Instituto de Ceramica y Vidrio, CSIC, C/Kelsen, n°. 5, Campus de Cantoblanco, Madrid, 28049 Spain; 3 Departamento de Física de Materiales Universidad Complutense de Madrid, Plaza de Ciencias 1, Ciudad Universitaria, Madrid, 28040 Spain

## Abstract

In this work, we demonstrate theoretically and experimentally the possibility of tuning the electromagnetic properties of metamaterials with magnetic fields by incorporating amorphous magnetic microwires. The large permeability of these wires at microwave frequencies allows tuning the resonance of the metamaterial by using magnetic fields of the order of tens of *Oe*. We describe here the physical basis of the interaction between a prototypical magnetic metamaterial with magnetic microwires and electromagnetic waves plus providing detailed calculations and experimental results for the case of an array of Split Ring Resonators with Co-based microwires.

## Introduction

Metamaterials are artificial patterned structures that possess unusual electromagnetic properties in a specific frequency band^1^. These exotic properties appear for radiation wavelengths (λ) fairly larger than the characteristic length of the microstructure lattice (*a*), that is, *a* ≪ λ. In these conditions, the wave cannot resolve the internal structure of the medium and a homogenous effective electric permittivity (*ε*_*eff*_) and magnetic permeability (*μ*_*eff*_) are enough to describe the electromagnetic properties of the medium^2^. A very important consequence is that metamaterials might exhibit a negative effective electric permittivity and/or a magnetic permeability at the microwave range^3^, leading to interesting properties as an opposite light refraction^4^ or an inverse Doppler effect^5^, with a wide range of applications^6–10^. All the properties of these artificial structures occur in a narrow frequency band, which results fixed by the geometric arrange of the array and the composition of the metamaterial. Therefore, there is an interest in developing tunable metamaterials, in order to switch and modulate electromagnetic waves by an external stimulus, i.e. for generating a patterned radiation^11^, selecting different work frequencies in imaging applications or in electromagnetic cloaking^12–14^. Different methods to tune the properties of metamaterials with external stimulus have been proposed such as electrical^15^, mechanical^16^ or optical stimulus^17^. These methods have accomplished experimental relative variations of the transmission and reflection spectra of different optical and microwave metamaterials^16, 17^ up to a 50% at their resonance. Effective shift of the resonant frequency results more complicated to achieve, but some tunable metamaterials in the Terahertz band are close to variations of their resonance frequency of several *THz* ^18^, or round 1 *GHz* in the case of microwave metamaterials^19^. Most of these achievements are based on the modulation of the electric properties of the metamaterial by means of their capacitance rather than variations in their intrinsic inductance^20^. Indeed, little research exists on tuning the basic properties of metamaterials using their magnetic properties. The main reason is that most of ferromagnetic materials are not active at the microwave band^21, 22^, being an exemption some ferrites. For example, Kang *et al*.^23^ have demonstrated the possibility of a magnetic tuning in a left-handed metamaterial using ferrite rods, accomplishing a frequency modulation of several Megahertz of the resonance frequency (10 *GHz* in their case) upon application of static magnetic fields of the order of 1000 *Oe*. This effect is based on the large variations of the magnetic permeability of YIG rods at high frequencies (that for GHz frequencies exhibit values of the permeability μ~14) when their ferromagnetic resonance is induced. Nevertheless, the use of ferrites in metamaterials can present some limitations: first, most of ferrite compounds exhibit a moderated magnetic permeability only close to their ferromagnetic resonance^24, 25^ limiting the working frequency; and secondly the effective variation of their magnetic permeability occurs for applied fields of thousands of *Oe*. For these reasons, the possibility of modulate the electromagnetic properties of a metamaterial with magnetic fields has been scarcely explored^26^.

An alternative approach to overcome this problem might be the incorporation of magnetic amorphous microwires exhibiting a high permeability at the microwave range^27, 28^ and presenting a soft magnetic behavior (saturation field lower than 20 *Oe*)^29, 30^. In particular, microwires based on Co exhibit almost null magnetostriction coefficients, and consequently they show a large magnetic permeability at the microwave range (ranging between 1–100 at the microwave range between 1 and 10 *GHz*)^31, 32^, giving rise to an outstanding magnetoimpedance effect^30^. Tunable metamaterials based on the incorporation of amorphous magnetic microwires, might exhibit significant advances in comparison with existing one. On the one hand, magnetic tuning does not need electrical contacts, eliminating, therefore, the possibility to induce electronic noise in the system from the source used to tune the metamaterial. On the other hand, the system can be tuned using moderated magnetic fields of the order of ~10 *Oe*, in comparison with the large magnetic fields required for the tuning of metamaterials based on ferrites.

In this paper, we demonstrate theoretically and experimentally the tuning of the electromagnetic properties of metamaterials at microwave frequencies with moderated DC magnetic fields by incorporation of Co-based magnetic microwires. In particular, we analyze here the case of an array of Split-Ring Resonators (SRR), but the method can be extended to many other structures with a negative magnetic permeability.

## Results

### Theoretical Model

**Figure 1:**
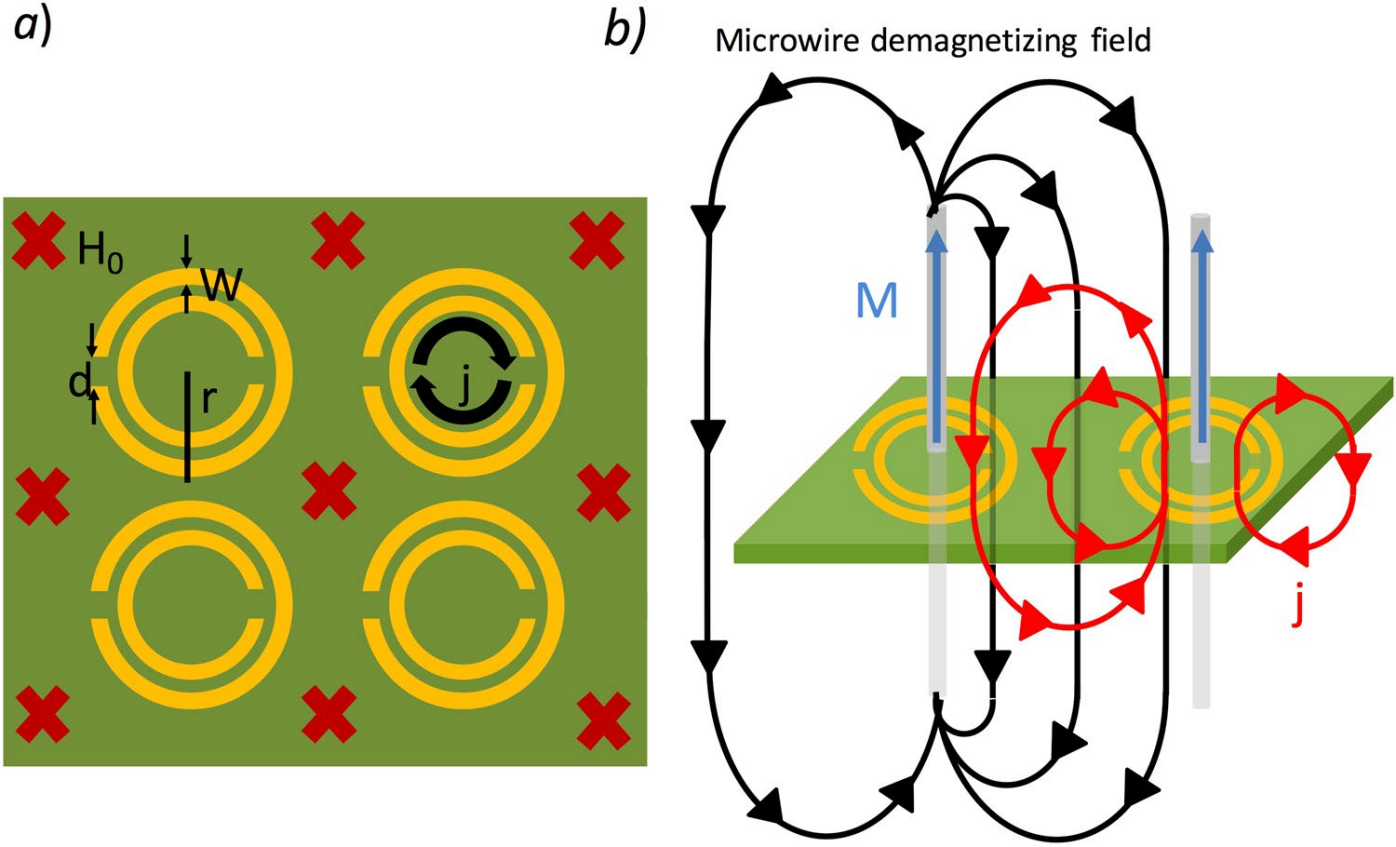
(**a**) Scheme of the studied and constructed array of SRR, where the external radius is about *3*.*6 mm*, the width of the rings is 0.*9 mm*, and the gap is about 0.2 *mm*. The oscillating applied field, *H*_*0*_, and the induced density current, *j*, are also represented. (**b**) Magnetic lines generated by a microwire when is included at the center of a SRR and the magnetic line representation of the field *j* and the demagnetiziting field.

The first metamaterial with a negative magnetic permeability at microwave frequencies was proposed by J.B. Pendry *et al*.^33, 34^ and consisted on a periodic array of Split-Ring Resonators (SRR) of external radius *r*: a C-shaped metallic structure composed by two concentric rings, each one with a gap in mutual opposition as shown in Fig. 1(a). Considering an electromagnetic wave, with a wavelength, *λ*, larger than the lattice parameter of the array, *a* ≪ *λ*, and a magnetic field, *H*_0_, parallel to the axis of the SRRs, a density current per unit length, *j*, is induced along the SRRs. The total field inside the SRR is the sum of the fields of the electromagnetic wave, *H*_0_, the field generated by *j*, and the demagnetizing field associated with the rest of SRRs in the array. The effective permeability of the system, *µ*_*eff*_, defined as the ratio between the magnetic field averaged in the unit cell, *B*_*ave*_ = *μ*_0_ *H*_0_, and *H*_*ave*_ can achieve negative values at certain frequency band that depends on the dimensions of the system.

Let us consider now a magnetic microwire (with radius *r*_*mw*_ ≪ *r* and length *l*) inserted at the center of each SRR with the magnetization parallel to the axis of the microwires as shown in Fig. 1(b). The presence of the magnetic wire introduces two additional magnetic fields in the array leading to an extra flux across the resonator: ${{\rm{\Phi }}}_{MW}={\mu }_{0}M(\pi {r}_{mw}^{2}-\alpha )$, where the first term is associated with the magnetization of the wire inside the cell; and the second term is due to the demagnetizing field arising from all the wires in the array that closes within the ring. For microwires much shorter than the radius of the resonator (*l* ≪ *r*), the *α* parameter is given by *α* = $\pi {r}_{mw}^{2}$, and no modification of the net flux across the resonator is induced by the presence of the microwire. On the other limit, if the length of the microwire is considered infinite, compared with the dimensions of the array, the parameter *α* goes to zero. We will focus here on this later case while the full analysis for any value of *α* is presented in the Supplementary Information. In the case of infinite microwires, the flux across the resonator is given by:

1 $${\rm{\Phi }}=\pi {r}^{2}{\mu }_{0}\,[{H}_{0}+j(1-\frac{\pi {r}^{2}}{{a}^{2}})+M\,(\frac{{r}_{mw}^{2}}{{r}^{2}})\,]$$

The electrical current flowing through the resonator to compensate the induced electromotive force is:

2 $$j=-\frac{{H}_{0}}{(1-\frac{\pi {r}^{2}}{{a}^{2}})+\frac{2\sigma i}{\omega r{\mu }_{0}\,(1+\frac{{r}_{mw}^{2}}{{r}^{2}}\chi )}-\frac{3}{\pi {\omega }^{2}{r}^{3}C{\mu }_{0}\,(1+\frac{{r}_{mw}^{2}}{{r}^{2}}\chi )}}$$

Where $\chi =\chi ({H}_{DC},\omega )$ is the differential susceptibility of the microwire for an AC magnetic field with frequency *ω* in presence of a DC magnetic field, *H*_*DC*_, *σ* and *C* are the resistivity per unit area and the capacity of the SRR, respectively.

The effective permeability of the metamaterial is defined as ${\mu }_{eff}=\frac{{B}_{ave}}{\,{\mu }_{0}{H}_{ave}}\,\,$where both *B*_*ave*_ and *H*_*ave*_ are AC magnetic fields at the frequency of the electromagnetic wave. These fields are obtained averaging over the faces and the lines of the unit cell respectively as described in ref. 33. *B*_*ave*_ is then given by:

3 $${B}_{ave}={\mu }_{c}{H}_{0}\,$$

being *μ*_*c*_:

4 $${\mu }_{c}={\mu }_{0}(1+\frac{\pi {r}_{mw}^{2}}{{a}^{2}}\chi )$$

On the contrary the value of *H*_*ave*_ is:

5 $${H}_{ave}={H}_{0}-\frac{\pi {r}^{2}}{{a}^{2}}j$$

Note that since *H*_*ave*_ is obtained averaging over lines out of the resonators and we are neglecting the demagnetizing field, the only modification introduced by the microwires is the variation of the current that flows over the surface of the rings. Moreover, note that setting *χ* = 0 we recover the original expressions for *B*_*ave*_ and *H*_*ave*_ of the SSRs in absence of microwires.

Using equations (3), (4), and (5), the effective magnetic permeability of the array plus the microwires is:

6 $${\mu }_{eff}=\frac{{B}_{ave}}{\,{\mu }_{0}{H}_{ave}}=\frac{{\mu }_{c}}{{\mu }_{0}}(1-\frac{1-\frac{{\omega }_{m0}^{2}}{{\omega }_{mP}^{2}}}{1+\frac{2\sigma i}{r\omega {\mu }_{0}{\mu }_{s}}-\frac{{\omega }_{m0}^{2}}{{\omega }^{2}}})$$

We define the parameters *μ*_S_, *ω*_*m0*_ and *ω*_*mP*_ by:

7 $${{\rm{\mu }}}_{{s}}={\mu }_{0}(1+\frac{{r}_{mw}^{2}}{{r}^{2}}\chi )$$

8 $${\omega }_{m0}=\sqrt{\frac{3}{{\pi }^{2}{r}^{3}{\mu }_{0}C\,(1+\frac{{r}_{mw}^{2}}{{r}^{2}}\chi )}}=\frac{{\omega }_{0}}{\sqrt{1+\frac{{r}_{mw}^{2}}{{r}^{2}}\chi }}$$

9 $${\omega }_{mP}=\sqrt{\frac{3}{{\pi }^{2}{r}^{3}{\mu }_{0}{\mu }_{s}(1-\frac{\pi {r}^{2}}{{a}^{2}})}\,}=\,\frac{{\omega }_{p}}{\sqrt{{\mu }_{s}}}$$

being *ω*_*0*_ and *ω*_*p*_ the resonant and plasma frequency of the metamaterial respectively in absence of microwires and *ω*_*m0*_ and *ω*_*mP*_ their counterparts in presence of microwires. Note that equations (6) and (8) demonstrate the possibility to tune the resonance of a metamaterial by modifying the differential susceptibility of the magnetic insertions. Actually, in our model the tuning is purely magnetic, while the application of the magnetic field does not alter the dielectric permittivity of the medium.

### Selection of the number of Microwires included in the metamaterial

Equation (6) demonstrates the possibility to tune the resonance of a metamaterial by modifying the differential susceptibility of the magnetic microwires. Thus, for a non-negligible tuning of the resonance the term $\frac{{r}_{mw}^{2}}{{r}^{2}}\chi (\omega ,{H}_{DC})$ in equations (6) and (8) must reach values comparable to 1 in absence of an external DC magnetic field (that corresponds to the maximum magnetic susceptibility). Since amorphous magnetic microwires typically have initial permeability of $\chi  \sim 1-100\,{\rm{at}}\,\mathrm{Gigahertzs}\,$ ^27, 28^ being its radius ~3·10^−5^ *m* and the SRR radius of a metamaterial working at *GHz* frequencies is ~*4* · *10* ^−*3*^ *m*, then for a single microwire, the factor $\chi \frac{{r}_{mw}^{2}}{{r}^{2}} \sim {10}^{-4}-{10}^{-3}$ is too low to induce a significant modification in the electromagnetic properties of the metamaterial. However, if we include *N* microwires per unit cell, the term $\frac{{r}_{mw}^{2}}{{r}^{2}}\chi$ in the above equations becomes $N\frac{{r}_{mw}^{2}}{{r}^{2}}$. For example, with the inclusion of 100 magnetic microwires the term $N\frac{{r}_{mw}^{2}}{{r}^{2}}\chi  \sim 0.01-0.1$, which allows tailoring the electromagnetic properties of the metamaterial upon moderated DC magnetic fields. Note that 100 microwires represent less than a 0.5% of the total volume of the unit cell of the metamaterial, so their presence do not induce significant limitations in the propagation of the microwaves or represent an increase of the metamaterial weight.

As above indicated, typical metallic ferromagnets (Fe or Ni) exhibit negligible permeability in the frequency range between 1 and 10 *GHz* ^35, 36^ rending impossible to use it for tuning the resonance of metamaterials. Thus, the unique properties of the amorphous magnetic microwires exhibiting large magnetoimpedance at microwaves frequency are the key for the magnetic control of the resonance of metamaterials. For these reasons, in the experiments were used 100 microwires that, as demonstrated above, will generate a noticeable switching effect in the absorption properties of the metamaterial.

### Magnetic characterization of the microwires

**Figure 2:**
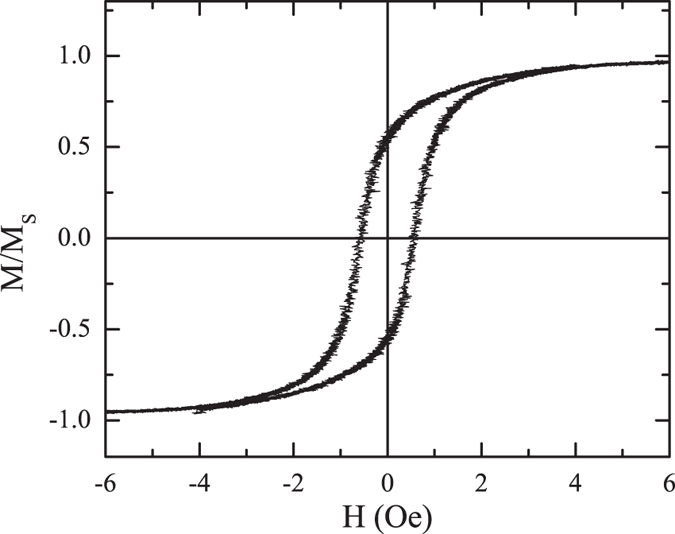
Hysteresis loop for one of the microwires used during the experiments obtained by induction method. The applied field was of 20 *Oe* of amplitude and 10 *Hz* of frequency.

Previous magnetic characterization of the used Magnetic Microwires in the experiment was conducted by a traditional induction method. The microwire composition was Fe_2.25_Co_72.75_Si_10_B_15_ with a nominal radius of 33 *μm* and length of 4 *cm* (more details are provided in the *Methods* section). The results, presented in Fig. 2, reveal a soft magnetic character of the microwires: with a coercive field less than 1 *Oe* and a saturation field of 4 *Oe* (the results are plotted in Fig. 2).

### SRR microwave characterization

**Figure 3:**
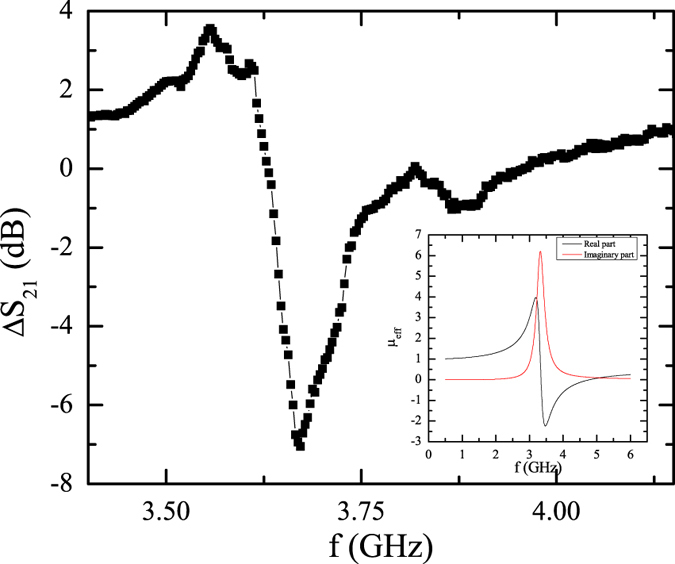
Scattering coefficient *S*_*21*_ (referenced to the spectrum without sample) measured for the array of SRR without microwires. The inset shows the theoretical effective magnetic permeability calculated according to equation (6) and χ = 0.

Initially, we measured the microwave spectrum of the metamaterial prior to the incorporation of the microwires. Figure 3 shows the variation of the scattering coefficient *S*_21_ provided by a Programmable Network Analyzer when the SRR array is placed between the emitted and receiving antennas. The spectrum (Fig. 3) exhibits a resonance at 3.55 *GHz* ^37, 38^. This frequency matches with the resonance of the theoretical permeability of the SSR array in the absence of the microwires (χ = 0 in equation (6) as shown in the inset of Fig. 3).

### Microwave characterization of the metamaterial composed by a SRR array plus magnetic microwires

**Figure 4:**
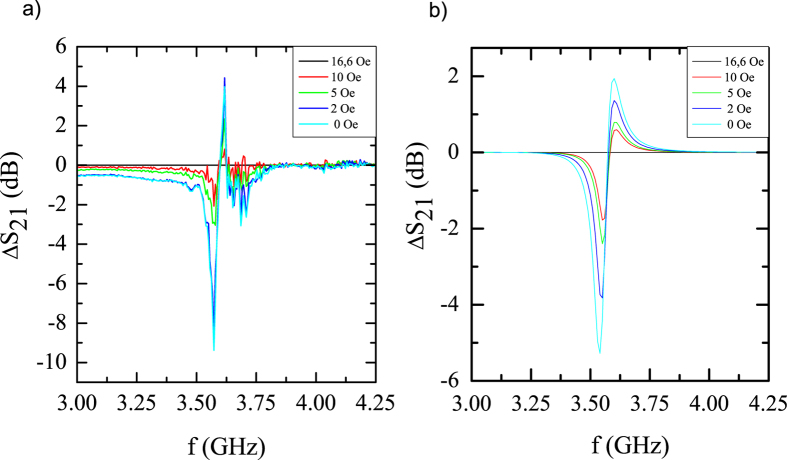
(**a**) Scattering coefficient (*ΔS*_*12*_) for the SRR metamaterial with 100 microwires per unit cell for different applied magnetic fields (curves referenced to the spectrum obtained with *H* = 16.6 *Oe*). (**b**) Computed scattering coefficient using equation (6) as a function of the microwave frequency at the same magnetic fields than the experiments.

Figure 4(a) presents the variation of the scattering coefficient *S*_21_ as a function of an applied DC magnetic field up to 16.6 *Oe* parallel to the axis of the microwires. All the spectra are referenced to the spectrum obtained at 16.6 *Oe* field, for comparison proposes, (original spectra are included in the Supplementary Information). The intensity of the scattering coefficient S_21_ round the resonance frequency exhibits variations of 10 *dB* for fields below 20 *Oe*, while no shift of the resonance frequency occurs beyond the resolution of our data.

The measured parameter *S*_21_ mainly provides information about the transmittance of the metamaterial. In order to determine if this variation is due to changes in the absorption or the reflection coefficient of the mematerial we measured also the coefficient *S*_11_ (basically it provides information about the reflection process) and its dependence on the applied magnetic field. The results (included in the Supplementary Information) showed the same trend than the parameter *S*_21_ confirming that the microwires tune the absorption of the metamaterial.

Figure 4(b) shows the computed values of the *S*_21_ coefficient using equation (6) (see Methods for further information), also referenced to its value at the saturation field of the Magnetic Microwires (that corresponds to χ = 0). The calculated and measured variation of the scattering coefficient shows a good agreement in terms of shape, scale and magnetic field dependence. Certainly, there are some quantitative differences that are associated with experimental issues. While the real device for the experiment contained 2 × 2 rings, that considered for the simulation was infinite (i.e., periodic boundary conditions). In addition, in the simulation, all the microwires are considered equivalents and parallel, while the 400 microwires used for the experiment had small length and orientation differences leading to a dispersion of the resonance conditions. Nevertheless, we consider the agreement good enough to confirm the validity of the proposed model: The amplitude of the S-parameter suffers a noticeable change (up to 6 *dB*), similar to that observed experimentally (up to 10 *dB*), and therefore confirming the validity of the proposed model.

### Effect of the Magnetic State of the microwires on the SRR electromagnetic properties

**Figure 5:**
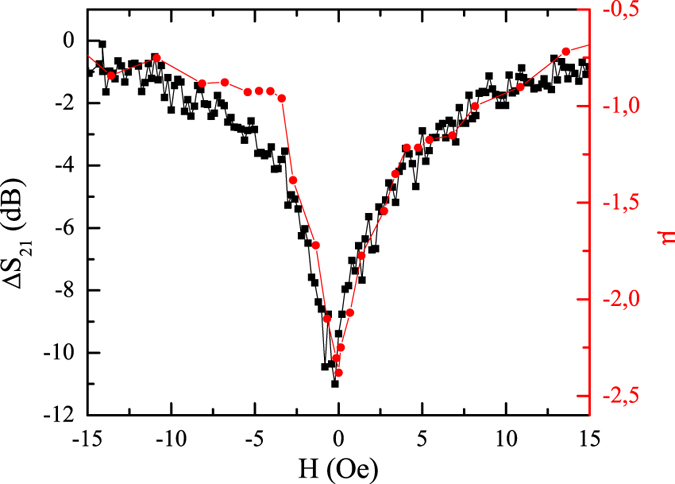
Comparission between the field dependence of the SRR array plus the microwires and the magnetic permeability of one of the microwires on the DC magnetic field. (Black squares) Dependence of the variation of the scattering coefficient at 3.55 *GHz* and (Red circles) of the magnetic permeability (with opposite sign) of one microwire at 3.75 *GHz* on the direct applied magnetic field.

The switching effect of the magnetic microwires on the metamaterial is also clearly seen when the minimum of the scattering coefficient *S*_21_ (at 3.55 *GHz*) in Fig. 4(b) is represented together with the measured magnetic permeability of one microwire (the measurement process is explained in the Mehotds section) as a function of the DC-magnetic field (Fig. 5). The experimental results reveal that the effect in the metamaterial is even on the magnetic field (i.e. equal for fields with the same intensity but opposite direction) and follows the same dependence on the magnetic field than the magnetic permeability of the microwires: The maximum variation of the minima occurs when the magnetic permeability does (round zero field), confirming again that the tuning of the resonance via DC magnetic fields is due to the variation of the magnetic state of the microwires. This result not only demonstrates the tuning effect of the magnetic microwires on the SRR array, but also confirms the physical mechanism of the tuning illustrated by equation (6). Therefore, the experimental results match with the parameters extracted from the simulations and the microwave characterization: Co-based magnetic microwires with non-negligible magnetic permeability at the microwave band induce significant changes at the resonance frequency for magnetic metamaterials through the modulation of their magnetic state by an external magnetic field. In particular, a switch effect is accomplished for variations of the direct field between 0 and 20 *Oe*.

## Discussion

We have shown here how the incorporation of magnetic microwires in a metamaterial based on a SRR array allows to manipulate its resonance with external DC magnetic fields. The underlying physical mechanism for this effect is the large magnetoimpedance of the microwires at *GHz* frequencies, which allows tuning their response via DC magnetic fields. In the particular device we studied here, upon magnetic fields of ~ 20 *Oe* the system achieves variations up to 10 *dB* in the absorbed power but larger effects can be achieved using other geometries and devices. This relative variation is a large value taking into account that our system consists in an array of 4 SRR. Therefore, we expect larger modulations for arrays with more elements, for which the absorption coefficient should exhibit values similar to previously reported tunable mematerials^20^ (round 30 *dB*).

In our experiments, we found a clear tuning of the intensity of the SRR array resonance. However, we did not observe tuning of the resonance frequency beyond the experimental resolution of our experimental set-up. The reason for that is the number of included microwires in the unit cell. From equation (8) the variation of the resonance frequency upon the inclusion of microwires in the SRR array is given by:

10 $$|\frac{{\omega }_{m0}-{\omega }_{0}}{{\omega }_{0}}|=1-\frac{1}{\sqrt{1+N\frac{{r}_{MW}^{2}}{{r}^{2}}\chi }}$$

Considering the mean radius of the microwires used during the experiment and the magnetic susceptibility measured for them at 3.75 *GHz*, for 100 wires included per unit cell the shift in frequency is a 0.5% (20 *MHz* for a work frequency of 3.55 *GHz*) that is below the resolution of our experimental set-up. This estimation perfectly matches with the performed simulations and with the feature that this shift is smaller than our experimental resolution. Nevertheless, larger shift of the resonance frequency can be accomplishes by increasing the number of microwires per unit cell. For instance, placing 10000 microwires per unit cell, yield a shift frequency of 1.75 *GHz*.

Those results point out that the inclusion of AMM provides a new method for magnetic tuning the resonance of metamaterials. In comparison with existing methods, our proposal has the advantage that the resonance can be tuned with magnetic fields of the order of ~10 Oe, although significant technological work needs to be done in order to achieve similar performance to existing methods. Remarkably, this method can be applied to tune the resonance of other type of metamaterials by considering the magnetic fields induced by the presence of the microwires as we described here.

## Methods

### Metamaterial fabrication

The 2 × 2 SRR array was fabricated by chemical etching in a plate of FR4 (relative permittivity of 3.84) and a copper layer of a thickness of 3.3 *μm*. The external radius of the split-rings was 3.6 *mm*, with width *w* = 0.9 *mm*, and gap *d* = 0.2 *mm*. The lattice parameter (i.e., distance between rings centers) was *a* = 8.8 *mm*. A hole was drilled at the center of each ring to include perpendicularly of the board 100 CoFeSi-based microwires with an outer radius of 33 *μm*.

### Characterizations

Magnetic characterization of the magnetic microwires was carried on a typical induction method with primary and secondary coils applying an ac-magnetic field parallel to the axis of the microwires of 20 *Oe* of amplitude and 10 *Hz* of frequency.

Microwave characterization of the samples was carried out by using two-printed dipolar antennas attached to a Programmable Network Analyzer (PNA from Agilent ltd, frequency range between 100 *MHz* and 20 *GHz*). The peak emission of the dipolar antennas was at 3.5 *GHz*, and the magnetic field of the wave was perpendicular to the SRR array. Two Helmholtz coils were used to applied DC-magnetic fields up to 16.6 *Oe* parallel to the axis of the microwires, in order to study the dependence of the microwave spectra on a the magnetic state of the microwire. A scheme of the complete experimental set-up is showed in the Fig. S1 of the Supplementary Information.

The magnetic permeability of the microwires at high frequency was studied inducing the resonance antenna of a 4 *cm* microwire length at 3.75 *GHz*. The experiment consisted in measuring the electric current induced through the microwires when the oscillating electric field of a linear antenna parallel to the axis of the microwire is applied. Therefore, one of the extremes of the microwire was attached to a Transmission Line and the other end to the ground of the line. The Transmission line was also connected to a Microwave Analyzer E7405A from Agilent ltd, working in the frequency range between 9 *kHz* and 26.5 *GHz*. The same Hemholtz coils, previously used in the microwave characterization, applied a direct field in order to study the dependence of the microwave permeability of the microwire on the DC field. The results are showed in the Supplementary Figure S4.

The electric current induced in a microwire of the same characteristics than the used on the experiments was computed using the Hallen-Pocklington equation^39^ as well as its dependence on the modulus of the magnetic permeability of the wire. Consequently, a direct conversion can be performed between the measured electric current and the modulus of the magnetic permeability.

### Calculus of the scattering coefficient S_21_

In order to compare the experimental results with the theoretical effective permeability deduced in equation (6), the scattering coefficients of an array of SRR within microwires was simulated using the effective permeability from equation (6). The method consists in computing the reflection, *Γ*, and transmission coefficient^40–43^, *T*, of the sample based on the effective permeability and permittivity of the studied medium. Then, the scattering coefficient, *S*_21_, is defined as:

11 $${S}_{21}=T\,\frac{1-{{\Gamma }}^{2}}{1-{{\Gamma }}^{2}{T}^{2}}$$

The transmission, and reflection coefficients, are related to the propagation constant, *γ*, and the impedance of the medium, *Z*, by:

12 $${\Gamma }=\frac{Z-{Z}_{0}}{Z+{Z}_{0}}$$

13 $$T={e}^{-\gamma d}$$

Where *d* is the thickness of the slab, and *Z*_0_ the vacuum impedance. The slab impedance, *Z*, and the propagation constant, *γ*, are related to the effective permittivity and permeability by:

14 $$Z=i\frac{\omega {\mu }_{0}{\mu }_{eff}}{\gamma }$$

15 $$\gamma =i\frac{2\pi f}{c}\sqrt{{{\epsilon }}_{eff}{\mu }_{eff}}$$

Equations (14) and (15) also apply for the vacuum case replacing the relative effective magnetic permeability and electric permittivity by 1. In our studies, the selected effective permeability for an array of SRRs within microwires was the same that for a SRRs array without microwires. This feature relies on the presence of the microwires affect to the magnetic behavior of the metamaterial, without introducing significant variations in the electric properties. Furthermore, in the field configuration in the experiments, the electric field of the microwave is perpendicular to the axis of the microwires, and hence the wires are not electrically excited. Therefore, the effective electric permittivity used in our calculus was:

16 $${{\epsilon }}_{eff}={(1-\frac{\pi {r}^{2}}{{a}^{2}})}^{-1}$$

As it is shown in equation (6) the effective permeability directly depends on the magnetic susceptibility of the magnetic microwires, so a similar dependence is expected on the scattering parameters. Using equation (6) and equations (11)–(16), the scattering coefficient ${S}_{21}(\chi ,\omega )$ was computed as a function of the microwire magnetic permeability, χ, and the frequency of the microwave, ω. In addition, to obtain a spectrum similar to the measure at the experiments, all the computed spectrums, ${S}_{21}(\chi ,\omega )$, were referenced respect the spectrum computed at zero susceptibility, because of in the experiments the reference spectrum was the saturate state of the microwires (at 16.6 *Oe*). In the theoretical analysis, the zero susceptibility spectrum was selected as reference because, in the saturated state of the microwires, the expected susceptibility is near zero. Therefore, the theoretical variation of S_21_ is defined as:

17 $${\Delta }{S}_{21}=20\,\mathrm{log}\,\frac{{S}_{21}\,(\chi ,\omega )}{{S}_{21}(\chi =0,\omega )}$$

The logarithm is taken to define the *S*_*21*_ in Decibels like in the experiments.

## Electronic supplementary material

Supplementary Information
