# Supplementary material for: Tuning Metamaterials by using Amorphous Magnetic Microwires
Source: Sci Rep. 2017 Aug 24;7:9394. doi: 10.1038/s41598-017-09665-5 (PMC5571162; doi:10.1038/s41598-017-09665-5)
Supplement: Supplementary file 1 — Supplementary Information [file 41598_2017_9665_MOESM1_ESM.pdf]

Supplementary Information for Tuning Metamaterials by using Amorphous Magnetic Microwires

V. Lopez-Dominguez[1,2,*], M.A. Garcia[1,2], P. Marin[1,3], and A. Hernando[1,3]

[1]Instituto de Magnetismo Aplicado UCM-adif, A6 km.22'5 – Apdo. Correos 155, Las Rozas, Madrid, 28230, Spain

[2]Instituto de Ceramica y Vidrio, CSIC, C/ Kelsen, nº. 5, Campus de Cantoblanco , Madrid, 28049,Spain

[3]Departamento de Física de Materiales Universidad Complutense de Madrid, Plaza de Ciencias, 1 Ciudad Universitaria, Madrid, 28040, Spain

S.1 Experimental set-up used for the microwave experiments

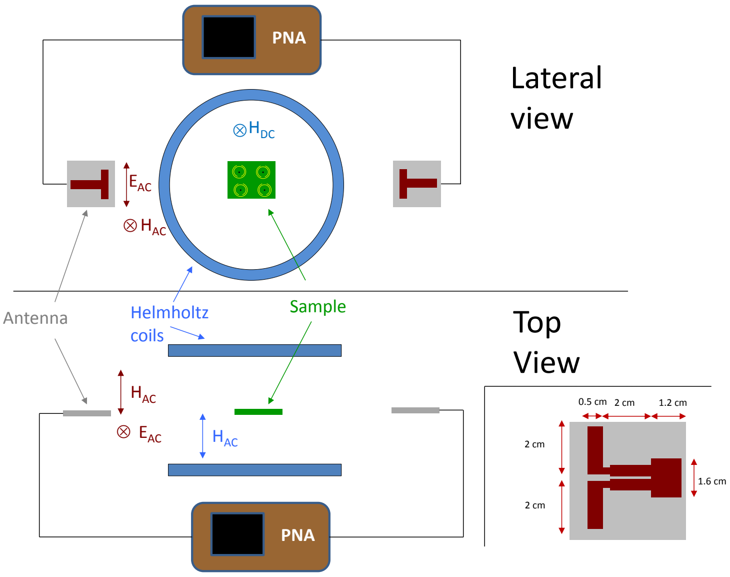

Figure S1. Scheme of the experimental set-up used for the Microwave experiments.

The experimental set-up consisted in a Programmable Network Analyzer (PNA) from Agilent Ltd working in the frequency range between 100 MHz and 20 GHz. Two coaxial cables are used to connect the PNA (ports 1 and 2) to two Cu homemade double dipolar antennas fabricated by chemical etching in an APPE substrate. The shape and dimension of the antennas are indicated in figure S1, and are optimized to emit at 3.5 *GHz*. The antennas are placed vertically emitting polarized waves with the electric field in the horizontal plane. The distance between the antennas

was 20 cm. The sample was placed between the antennas using a microwave transparent and diamagnetic support. Two Helmholtz coils (R= 16 *cm*) are used to apply a homogeneous DC magnetic field in the sample region, between -20 and 20 *Oe*.

S.2 Raw spectra measured by the scattering coefficient S_{21}

Fig. S.2 depicts the raw spectra measured for the SRR array and the magnetic microwires as a function of the applied DC magnetic fields. As the results reveal, the magnetic field changes the resonance peak of the SRR metamaterial, but these variations are difficult to observe in the raw data. For this reason in the manuscript, the spectra are referenced to the spectrum recorded at 16.6 *Oe*, to observe the modulation of the absorption peak due to the application of a DC magnetic field.

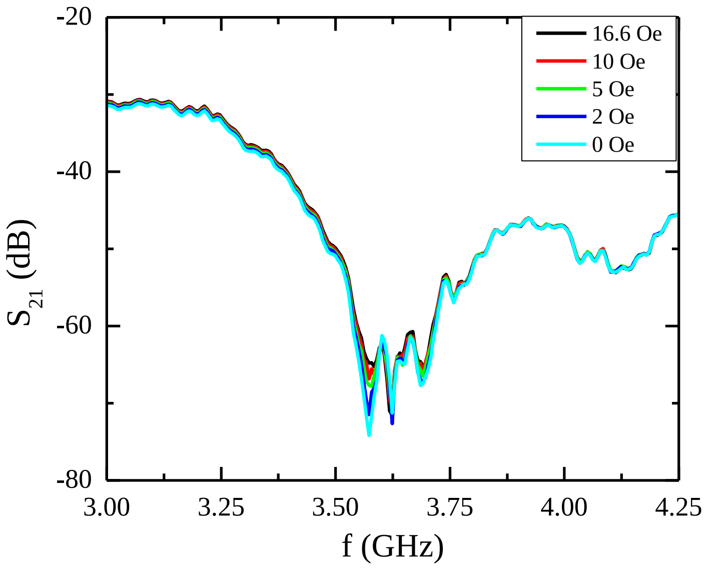

Figure. S.2. Raw spectra measured for the SRR array and the magnetic microwires as a function of the applied magnetic field.

S.3 Effective tuning of the Metamaterial plus the microwires measured by the scattering coefficient S_{11}

The microwave spectrum of the SRR array plus the magnetic microwires was also measured by means of the scattering coefficient S_{11}. As in the case of the S_{21} coefficient, each spectrum was

referenced to the spectrum measured at 16.6 *Oe* in order to study only the variations introduced by the magnetic state of the magnetic microwires.

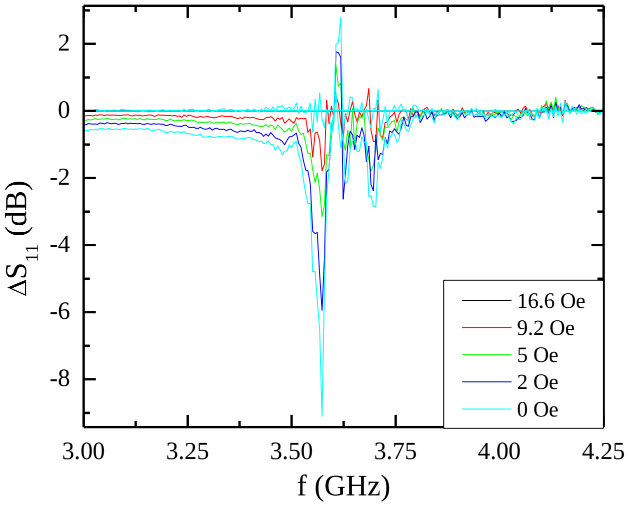

Figure S.3. Magnetic tuning by the magnetic microwires measured by way of the scattering coefficient S_{11}. Variations of the scattering coefficient S_{11} at different magnetic fields. All the spectra are referenced by the spectrum recorded at 16.6 *Oe*.

As the results reveal the same trend is observed than in the case of the scattering coefficient S_{21} showed in the main manuscript.

S.4 Induced electric current and magnetic permeability at the antenna resonance of a magnetic microwire

The electric current induced in the microwires upon illumination with 3.75 *GHz* microwaves is represented in Fig. S.4. The maximum electric current (Maximum Magnetoimpedance) occurs at zero field, that corresponds to the maximum values of the magnetic permeability. Moreover, for fields larger than the saturation field, H>4 *Oe* the current remains constant.

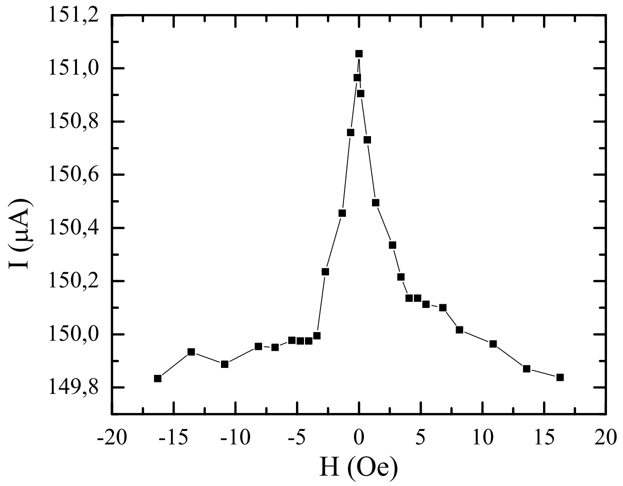

Figure S.4. Induced electric current measured along a magnetic microwire measured at the antenna resonance. Electric current directly measured for a 4 *cm* microwire length at 3.75 *GHz* applying different dc-magnetic fields parallel to the axis of the microwire.

S.5 Comparison of the microwave spectra between the microwires and the SRR array

The same microwave experiments realized to the SRRs, it described in the main manuscript, were also conducted for a set of 100 microwires, without the SRR array, of the same length used in the experiments (4 *cm*). As in the case of the SRR array, the microwave magnetic field, H_0, was parallel to the axis of the microwires, so the thin antenna resonance was not excited[1]. Furthermore, different spectra were recorded applying a DC magnetic field parallel to the axis of the microwire such as in the experiments with the SRR array. For direct comparison, each spectrum was referenced by the spectrum recorded at 16.6 *Oe.* In Fig. S.5 is represented the spectrum at 0 *Oe* and the same spectrum when the microwires are inserted in the SRR array.

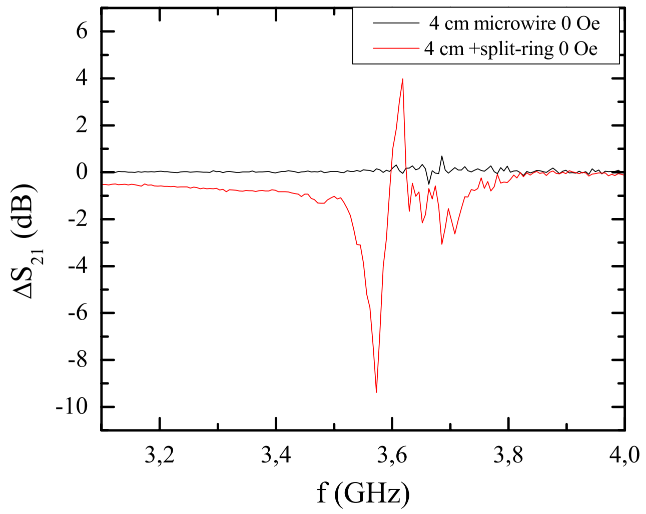

Fig. S.5. Microwave spectrum of 100 microwires compared with the spectrum of the SRR array plus the wires. Recorded spectrum at 0 *Oe* for a set of 100 magnetic microwires (black line), and the same spectra when a the microwires are included in a SRR array (red line).

As it is clearly seen in Fig. S.2, the spectrum of the isolated microwires is not modulated by the application of a magnetic field, whereas when they are included inside an SRR array their magnetic state modulates the response of the metamaterial at the resonance frequency of the array confirming that the observed resonance is due to the SSRs array**.**

S.6 Theoretical deduction of the effective permeability for an array of Split Ring Resonators within Magnetic Microwires

If we consider an array of Split Ring Resonator (SRR) of radius r [1] and we include a magnetic microwire (radius $r_{MW}<r$ and length l) placed at the center of each SRR, two new contributions have to be considered in the magnetic flux across the resonator: the first one associated with the magnetization of the microwire, M, and the second to the demagnetizing field of the microwire. Therefore, in presence of the microwire, the flux across the resonator is given by:

$$\Phi_{SRR} = \mu_0 \pi r^2 \left[H_0 + j\left(1 - \frac{\pi r^2}{a^2}\right) + M(\beta - \alpha) + H_{DC} \right] \quad (1)$$

where μ_0 is the vacuum magnetic permeability, $\beta = r_{MW}^2/r^2$ refers to the feature that the flux of the magnetization only occurs at the cross section of the wire, α is a parameter that takes into account the fraction of the demagnetizing field of the microwire that is enclosed within the resonator, and H_{DC} is a dc applied magnetic field along the microwire axis. The values of the parameter α ranges from zero to $\pi r_{mw}^2/r^2$ as described in the manuscript. The first case corresponds when the length of the microwire can be considered as infinite length in comparison with the dimension of the array. In this case, the part of the demagnetizing field of all the microwires that closes within the array can be neglected. On the other hand, the last case corresponds when the length of the microwire is much shorter than the radius of the SSRs. All the demagnetizing field is closed within the SSR and the net magnetic flux introduced by the microwire is null, $\alpha = \pi r_{mw}^2/r^2$. While in the manuscript we considered the case $\alpha=0$, we describe here the more general case for any value of α. The time variation of the magnetic flux depicted by equation (1) generates an electromotrive force in the SRR of the form:

$$\frac{d\Phi}{dt} = -\mu_o \pi r^2 \frac{\partial}{\partial t}\left[H_0 + j\left(1 - \frac{\pi r^2}{a^2}\right) + M(\beta - \alpha) + H_{DC}\right] \quad (2)$$

Since the oscillating fields in this system are small, the time derivative of the magnetization can be expressed in terms of the differential magnetic susceptibility:

$$\frac{dM}{dt} = \frac{\partial M}{\partial H}\frac{dH}{dt} = \chi(H_{DC},\omega)\frac{d}{dt}\left[H_0 + j\left(1 - \frac{\pi r^2}{a^2}\right) - \alpha M + H_{DC}\right] =$$

$$= \chi(H_{DC},\omega)\left\{-i\omega\left[H_0 + j\left(1 - \frac{\pi r^2}{a^2}\right)\right] - \alpha\frac{dM}{dt}\right\} \quad (3)$$

Where $\chi(H_{DC},\omega) = \frac{\partial M}{\partial H}$ holds for the differential magnetic susceptibility for an AC field with frequency ω and in presence of a DC field, H_{DC}. The total field in equation (3) is the local magnetic field at the position of the microwire, which is the external field, H_0 plus the induced field, j, and the demagnetizing field associated with the SRR and the microwires of other unit cells. Using equation (3) the time derivative of the magnetization is:

$$\frac{dM}{dt} = -i\omega\frac{\chi(H,\omega)}{1 + \alpha\chi(H,\omega)}\left[H_0 + j\left(1 - \frac{\pi r^2}{a^2}\right)\right] \qquad (4)$$

In addition, the magnetic susceptibility of the wire is considered fixed by the DC magnetic field and the wave frequency; $\chi(H_{DC}, \omega)$. Taking into account equations (3) to (4)

$$efm = i\omega\mu_S \pi r^2 \left[H_0 + j\left(1 - \frac{\pi r^2}{a^2}\right)\right] - 2\pi r\sigma j + \frac{Q}{C} = 0 \quad (5)$$

Being $\mu_s = \mu_0\left[1 + \frac{\chi}{1+\alpha\chi}(\beta - \alpha)\right]$, σ the resistivity of the SRR, *C* the electric capacity of the SRR, and *Q* the total charge. Equation (5) clearly shows the electromotrive force inside the SRR is essentially the same that the case without wires obtained by Pendry et al. [1], but the magnetic permeability of the vacuum is replaced by the new permeability μ_S. For example, for an infinity wire, $\alpha = 0$, the magnetic permeability is $\mu_s = \mu_0(1 + \beta\chi)$, which corresponds to the case studied in the main manuscript. From equation (5) the density current is computed, leading to:

$$j = \frac{-1}{\left(1 - \frac{\pi r^2}{a^2}\right) + \frac{2\sigma i}{\omega r \mu_s} - \frac{3}{\pi^2 \mu_s \omega^2 C r^3}} H_0 = F H_0 \quad (6)$$

Where *F* is given by

$$F = -\frac{1}{\left(1 - \frac{\pi r^2}{a^2}\right) + \frac{2\sigma i}{\omega r \mu_s} - \frac{3}{\pi^2 \mu_s \omega^2 C r^3}} \quad (7)$$

The average field H_{ave} is computed in a line outside the surface that encloses the SRR [1]:

$$H_{ave} = H_0 - \frac{\pi r^2}{a^2} j - \alpha M_{MW} = H_0 - \frac{\pi r^2}{a^2} j - \frac{\alpha\chi}{1+\alpha\chi}\left(H_0 - \frac{\pi r^2}{a^2} j\right) =$$

$$= A H_0 - B\frac{\pi r^2}{a^2} j \quad (8)$$

where the parameters *A* and *B* are defined as:

$$A \equiv 1 - \frac{\alpha\chi}{1+\alpha\chi} \quad (9)$$

$$B \equiv 1 - \frac{\alpha\chi}{(1+\alpha\chi)\frac{\pi r^2}{a^2}} \quad (10)$$

The magnetic field B_{ave} is averaged at the surfaces of the unit cell. In absence of microwires, the average value of the fields j and $j\pi r^2/a^2$ are zero, so that $B_{ave} = \mu_0 H_0$. However, the magnetization of the wire only occurs in the region enclosed by the radius of the wire. Therefore, the magnetic field of the microwire averaged outside the SRR surface is bM_{MW}, where $b \equiv \pi r_{MW}^2/a^2$. The average of the demagnetizing field will be $-\alpha^* M$, where the coefficient α^* ranges between the values 0 and $\frac{\pi r_{MW}^2}{a^2}$, that is in the whole cell. It is important to take into account that the coefficient α^* is different from the coefficient α used to define the demagnetizing field of the wires in equation (1). Thus the average field B is:

$$B_{ave} = \mu_0 H_0 + \mu_0 M_{MW}(b-\alpha^*) = \mu_0\left[1 + \frac{\chi\left(1 + F\left(1 - \frac{\pi r^2}{a^2}\right)(b-\alpha^*)\right)}{1+\alpha\chi}\right] H_0 =$$

$$= \mu_c H_o \quad (11)$$

being μ_c:

$$\mu_c \equiv \mu_0 \left\{1 + \frac{\chi\left[1 + F\left(1 - \frac{\pi r^2}{a^2}(b-\alpha^*)\right)\right]}{1+\alpha\chi}\right\} \quad (12)$$

Thus, we find that the effect of the magnetic microwires in the average field is equivalent to assume that the whole array is embedded in a medium of magnetic permeability $\mu_{c.}$ As indicated above, the oscillating fields are fairly smaller than the applied fields, H_0 and H_{DC}, then:

$$H_0 \gg j\left(1 - \frac{\pi r^2}{a^2}\right) = FH_0\left(1 - \frac{\pi r^2}{a^2}\right) \quad (13)$$

and $F\left(1 - \frac{\pi r^2}{a^2}\right) \ll 1$. Note that with this approximation and the infinite length wire, equation (11) turns into:

$$B_{ave} = \mu_0(1 + b\chi) \quad (14)$$

which corresponds to the result exposed in the manuscript.

Finally, the effective permeability of the metamaterial can be computed from the average fields:

$$\mu_{eff} = \frac{B_{ave}}{\mu_0 H_{ave}} = \mu_c' \left(1 - \frac{\frac{\pi r^2}{a^2}}{1 + \frac{2\sigma i}{\omega r \mu_S} - \frac{3}{\pi^2 r^3 \omega^2 C \mu_S}} \right) \qquad (15)$$

Where $\mu_c' = \mu_c/\mu_o$. This effective permeability exhibit a resonance for the frequency:

$$\omega_{m0} = \sqrt{\frac{3}{\pi^2 r^3 \mu_0 C \mu_s}} \qquad (16)$$

This resonance can be tuned applying suitable magnetic fields due to the dependence of μ_S on the magnetic susceptibility of the microwire. Finally the effective permeability deduced in equation (15) can be expressed as a function of the resonance frequency showed in equation (16) and the magnetic plasma frequency, defined as $\omega_{mp} = \sqrt{\frac{3}{\pi^2 r^3 \mu_0 \mu_s \left(1 - \frac{\pi r^2}{a^2}\right)}}$.

$$\mu_{eff} = \mu'_c \left(1 - \frac{1 - \frac{\omega_{m0}^2}{\omega_{mP}^2}}{1 + \frac{2\sigma i}{r\omega\mu_0 \left(1 + \frac{r_{MW}^2}{r^2}\chi\right)} - \frac{\omega_{m0}^2}{\omega^2}} \right) \qquad (17)$$

References

1. A. Hernando, V. Lopez-Dominguez, E. Ricciardi, K. Osiak, and P. Marin, IEEE Transactions on Antennas and Propagation **64**, pp. 1112-1115 (2016).
